# Clinical usefulness of splanchnic oxygenation in predicting necrotizing enterocolitis in extremely preterm infants: a cohort study

**DOI:** 10.1186/s12887-023-04145-4

**Published:** 2023-07-01

**Authors:** E. Palleri, M. van der Heide, J. B.F. Hulscher, M. Bartocci, T. Wester, E. M.W. Kooi

**Affiliations:** 1 Department of Neonatology, Karolinska University Hospital, Stockholm, Sweden; 2 Department of Women’s and Children’s Health, Karolinska Institutet, Stockholm, Sweden; 3 Division of Neonatology, University of Groningen, University Medical Center Groningen, Beatrix Children’s Hospital, Groningen, The Netherlands; 4 Department of Surgery, Division of Pediatric Surgery, University of Groningen, University Medical Center Groningen, Groningen, The Netherlands; 5 Department of Pediatric Surgery, Karolinska University Hospital, Stockholm, Sweden

**Keywords:** Necrotizing enterocolitis, Near infrared spectroscopy, Extremely preterm neonate

## Abstract

**Background:**

Impaired intestinal microcirculation seems to play an important role in the pathogenesis of necrotizing enterocolitis (NEC). A previous study showed that a SrSO_2_ < 30% is associated with an increased risk of developing of NEC. We aimed to determine the clinical usefulness of the cut off < 30% for SrSO_2_ in predicting NEC in extremely preterm neonates.

**Methods:**

This is a combined cohort observational study. We added a second cohort from another university hospital to the previous cohort of extremely preterm infants. SrSO_2_ was measured for 1–2 h at days 2–6 after birth. To determine clinical usefulness we assessed sensitivity, specificity, positive and negative predictive values for mean SrSO_2_ < 30. Odds ratio to develop NEC was assessed with generalized linear model analysis, adjusting for center.

**Results:**

We included 86 extremely preterm infants, median gestational age 26.3 weeks (range 23.0-27.9). Seventeen infants developed NEC. A mean SrSO_2_ < 30% was found in 70.5% of infants who developed NEC compared to 33.3% of those who did not (p = 0.01). Positive and negative predictive values were 0.33 CI (0.24–0.44) and 0.90 CI (0.83–0.96), respectively. The odds of developing NEC were 4.5 (95% CI 1.4–14.3) times higher in infants with SrSO2 < 30% compared to those with SrSO2 ≥ 30%.

**Conclusions:**

A mean SrSO_2_ cut off ≥ 30% in extremely preterm infants between days 2–6 after birth may be useful in identifying infants who will not develop NEC.

**Supplementary Information:**

The online version contains supplementary material available at 10.1186/s12887-023-04145-4.

## Introduction

Impaired microcirculation of the intestines seems to play an important role in the pathogenesis of necrotizing enterocolitis (NEC) in extremely preterm neonates [1]. Fetuses and newborn animals have an immature splanchnic perfusion, with a less pronounced vasodilatory response and an increased oxygen extraction to systemic stress, when compared with older animals and humans [2, 3]. Similarly, many studies on intestinal perfusion measured by near-infrared spectroscopy (NIRS) have reported that splanchnic oxygenation is impaired before the onset of NEC in preterm infants [4, 5].

Since bowel ischemia, or at least impaired intestinal perfusion, seems to be associated with the development of NEC [1, 6], measuring splanchnic oxygenation (SrSO_2_) and oxygen extraction might detect changes compatible with the pre-stages of NEC. Piglet models have shown that low SrSO_2_ within the first days of life is a risk factor for NEC [7] and, indeed, impaired SrSO_2_ before the onset of clinical NEC has been reported in preterm infants (3, 4).

In a previous study in extremely preterm infants, it was shown that a 1-hour mean splanchnic oxygenation < 30%, between days 2 and 6 of life, was associated with an increased risk of developing NEC, but this risk seems to change through gestational age [8]. In order to understand the validity and utility of our previous findings given the relatively low samples size, we chose to combine our cohort with another comparable European cohort to study the clinical usefulness of the splanchnic oxygenation cut off. The aim of this study was to strengthen our previous findings in a larger pooled cohort and to determine whether the cut off value of < 30% for mean SrSO_2_ in the first week after birth has some clinical usefulness and can predict NEC in extremely preterm neonates.

## Methods

This was a combined cohort study pooling extremely preterm neonates (< 28 weeks gestational age) from two level III NICUs at the Karolinska University Hospital, Stockholm, Sweden and the University Medical Center, Groningen, Netherlands.

### Participants

The population in this study was formed by merging two European cohorts (one Swedish and one Dutch) of extremely preterm newborns where cerebral and splanchnic monitoring was performed in the first week of life. Inclusion criteria were: infants born before 28 weeks of gestation. Exclusion criteria were infants born before 23 weeks of gestation, major abdominal wall defects, major known intra-abdominal malformations, infants who already had NEC/ spontaneous intestinal perforation or developed NEC/ spontaneous intestinal perforation within 24 h from the measurements and critically ill neonates not tolerating abdominal ultrasound or NIRS monitoring.

### Setting

#### Swedish cohort

Extremely preterm infants, born before 28 weeks of gestational age, were included in the study from September 2014 to December 2016. Cerebral and splanchnic oxygenation monitoring was performed at postnatal age between days 2 and 6, within 96 h after the first enteral feeding. This population has been described in detail in a previous publication [8]. A near infrared spectroscopy (NIRS) INVOS 5100c monitor (Medtronic™, (Minneapolis,) USA) with neonatal INVOS sensors was used to measure cerebral (CrSO_2_) and splanchnic regional oxygenation (SrSO_2_). To measure cerebral oxygenation, a sensor was placed in the frontoparietal side of the head. For the measurement of splanchnic oxygenation, a sensor was placed just below the umbilicus. For each NIRS recording we allowed a baseline of 5 to 15 min for stabilization, which was not included in the analysis. The measurements were performed for at least one hour. According to the local feeding protocol, infants with birth weights < 1250 g were fed by continuous enteral feeds and > 1250 g by bolus feeds during the measurements.

#### Dutch cohort

Extremely preterm infants, born before 28 weeks of gestational age, were included in the study from January 2016 to March 2018. With the same monitor as the Swedish cohort, INVOS 5100c monitor (Medtronic™, (Minneapolis,) USA), we measured cerebral and splanchnic oxygenation between days 1 and 7 after birth as part of standard clinical care. The neonatal INVOS™ SomaSensor was placed below the umbilicus and on the left or right frontoparietal side of the head for the measurement of SrSO_2_ and CrSO_2_, respectively. Sensors were placed on top of Mepitel® sheets (Mölnlycke, Sweden) for skin protection [9]. Data were saved in an offline pseudonymized database.

For research purposes the sensor placement was checked and verified every morning by the attending nurse and researcher. A daily two-hour mean of rsSO2 was calculated before or, when unavailable, closest to the sensor verification. A 2-hour period with at least 80% of available data was selected. According to local protocol, every infant received intermittent bolus feeding every two hours. We selected the measurements at day 4 or, when unavailable, at day 3 or 5 for the comparison with the Swedish cohort.

### Clinical variables

Clinical variables collected from the infants’ charts included: demographic data, small for gestational age status (according to the national reference values)[10, 11], amount and type of feeding, hemoglobin levels, presence of intraventricular hemorrhage, Apgar score at 5 min, respiratory support, hemodynamically significant patent ductus arteriosus (defined as infants needing treatment), postnatal age at time of NIRS measurement and mortality.

The outcome was the *NEC diagnosis*, which was based on clinical signs plus the presence of intramural gas on abdominal radiographs and/or histological evidence of NEC. In both cohorts, NEC diagnosis was verified by the authors of the site (EP,MB,EK,JH) according to the Bell’s criteria. Unclear cases were discussed together, and consensus was reached in all cases. Infants with only a clinical suspicion of NEC (Bell´s stage < 2 A) were not considered as having NEC. Surgical NEC was defined as a patient who had an indication for surgical treatment according to the treating physicians.

### Data and statistical analysis

NIRS data were collected automatically once every 6 s and stored offline for analysis. The data were analyzed for artifacts which afterwards were removed. In both cohorts, artifacts were defined as: changes in SrSO_2_ and CrSO_2_ that could not be physiologically explained, misplacement of the sensor or missing rSO2 values due to measurement failure. We chose not to exclude the 15% and 95% values (the minimum and maximum values for the device used) from our analyses.

Regarding NIRS variables, we calculated the mean SrSO_2_ and CrSO_2_. Moreover, we calculated the splanchnic-cerebral oxygenation ratio (SCOR) as splanchnic oxygenation divided by mean cerebral oxygenation (SrSO_2_/CrSO_2_).

First, demographic and clinical variables were compared between the two cohorts. Second, we divided all infants based on the SrSO_2_ 30% cut off [8]. Third, specificity, sensitivity, and positive and negative predictive values with confidence intervals were calculated with a bootstrap cross-validation in R 4.1.2. Fourth, generalized linear models were applied, with logit link and NEC as dependent variable, to analyze the primary outcome (NEC) which was presented as an odds ratio. Finally, variables for the final model were chosen through univariate analysis with a P-value < 0.05.

The Mann-Whitney *U* test, Student’s t-test and *chi*^2^ or Fisher‘s exact test were used for non-parametric, parametric, or categorical data, respectively. We used the statistical program STATA version 14.2 (StataCorp, TX) to perform the rest of the analyses.

## Results

### Participants

**Fig. 1:**
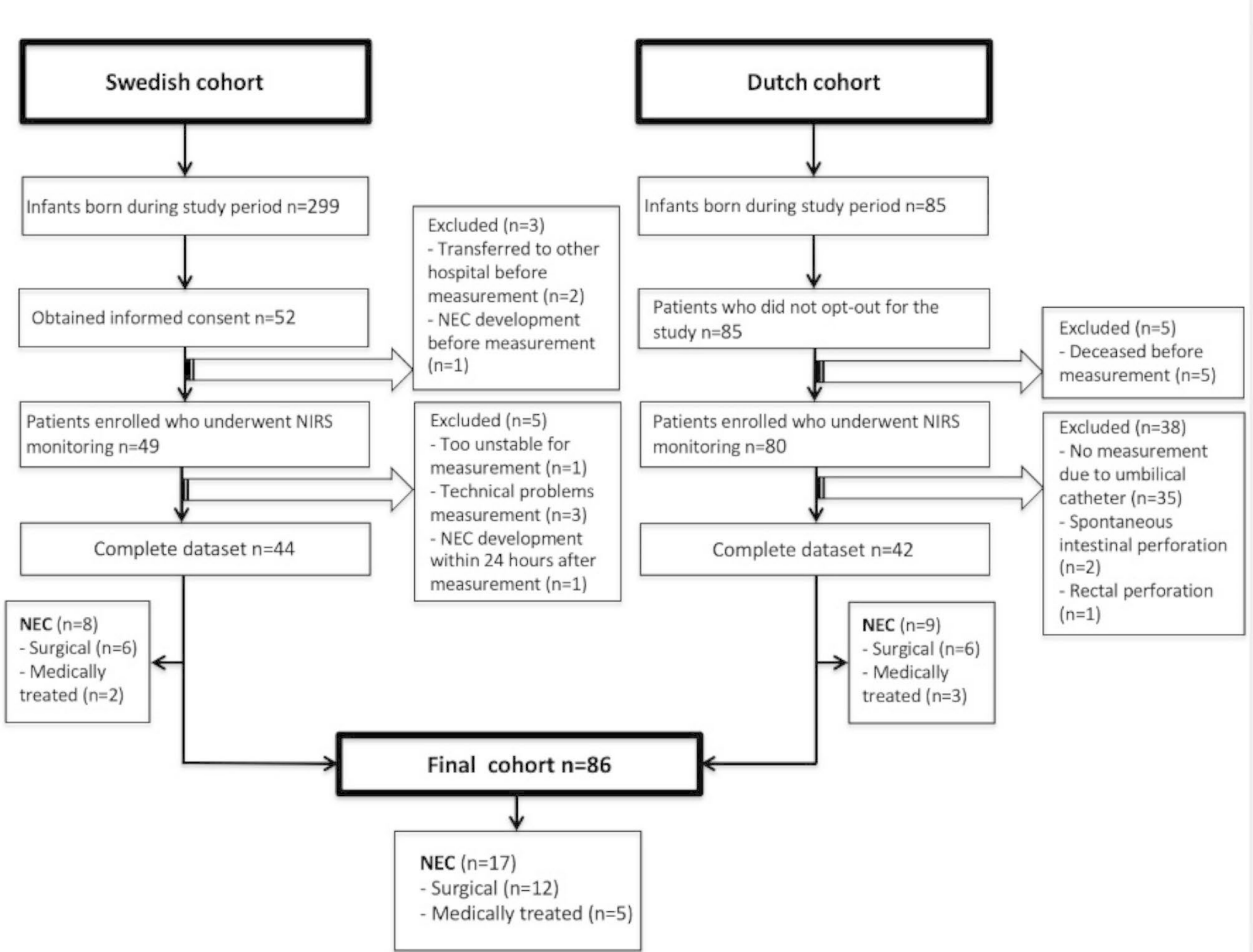
Flow chart (Technical issues include: unavailable research team, poor quality of NIRS registration with too many artefacts, uncomplete NIRS data because of storage problems)

Eighty-six extremely preterm infants were included in the analysis: 44 from the Swedish and 42 from the Dutch cohort (Fig. 1). Eight infants developed NEC in the Swedish and nine in the Dutch cohort, the median postnatal age at NEC diagnosis was 15 (IQR 10–23) days. The incidence of NEC was 19.8%, with 70% of the NEC cases requiring surgical intervention.

Patient characteristics of the two cohorts are shown in Table 1. Median gestational age of the final total cohort was 26.3 weeks IQR (25.3–27.1). Infants from the Swedish cohort had significantly lower gestational age, lower birth weight, and were more often delivered by caesarean section. All infants underwent NIRS monitoring at a mean postnatal age of 4.0 ± 0.9 days. Median SrSO2 was 34.8 (21.5–52.4)%. There was no difference in median SrSO_2,_ CrSO_2_ or SCOR between the two centers.

**Table 1:** Patients characteristics

	Dutch cohort (n = 42)	Swedish cohort (n = 44)	Combined cohort (n = 86)
Male, n(%)	26 (61.9)	27 (61.4)	53 (61.6)
Gestational age (weeks), (median (IQR))	26.9 (26.1–27.4)	25.6 (24.3–26.5)	26.3(25.3–27.1)
Caesarean section, n(%)	11 (26.2)	34 (77.3)	45 (52.8)
APGAR score at 5 min, (median (IQR))	7 (6–8)	7 (5–8)	7(6–8)
Small for gestational age, n(%)	8 (19.1)	8 (18.2)	16 (18.6)
Birth weight (grams) (mean (SD))	929 (± 277)	739 (± 173)	832 (± 247)
IVH≥2, n(%)	5 (11.9)	6 (13.6)	11 (12.8)
hsPDA, n(%)	28 (66.7)	22 (50)	50 (58.1)
Postnatal age at NIRS measurement (days) (mean (SD)	3.92 (0.41)	4.10 (1.21)	4.02 (0.91)
Hemoglobin g/L at NIRS* (median (IQR))^a^	143.4 (128.9-156.3)	141 (135-148.5)	142 (134.0-153.1)
Mechanical ventilation, n(%)	17 (40.5)	21 (47.7)	38 (44.2)
feeding (ml/kg/day) > 30 ml/kg/d, n(%)	26 (61.9)	32 (72.7)	58 (67.4)
exclusive breast milk at NIRS measurement, n(%)b	18 (45.0)	44 (100.0)	62 (73.8)
> 50% breast milk at NIRS measurement, n(%)b	37 (92.5)	44 (100.0)	81 (96.4)
SrSO2 (median(IQR))	30.4 (19.8–52.0)	37.9 (25.0-53.4)	34.83 (21.5–52.4)
SrSO2 < 30, n(%)	20 (45.2)	16 (36.4)	36 (41.8)
CrSO2,median(IQR)	77.3(70.5–82.4)	75.7(70.1–80.6)	76.7(70.2–81.8)
SCOR, median (IQR)	0.39 (0.28–0.65)	0.50 (0.33–0.76)	0.47 (0.30–0.73)
NEC, n(%)	9 (21.4)	8 (18.2)	17 (19.8)
Surgical NEC, n(%)	6 (14.3)	6 (13.6)	12 (13.9)

Data are expressed as median (IQR = interquartile range), mean ± standard deviation for continuous variables or as number (percentage) for categorical variables. UMCG: University Medical Center Groningen. IVH: Intraventricular hemorrhage. hsPDA: hemodynamically significant patent ductus arteriosus. NEC: necrotizing enterocolitis. a:missing data for 3 patients. b: missing data for 2 patients

### Outcomes: Splanchnic oxygenation cut off and NE

In the final cohort, 36 (41.8%) infants had a mean SrSO_2_ < 30% between days 2–6 of life. These infants had a higher incidence of NEC (33.3% vs. 10.0%, p = 0.012) and surgical NEC (27.8% vs. 4%, p = 0.003) than infants with a SrSO_2_ ≥ 30% (Table 2). The median age at NEC was 15 day (IQR 10–23). We did not find any other differences between the two groups (Table 2). Of all infants who later on developed NEC, 70.6% had a SrSO_2_ < 30% while only 33.3% of infants who did not develop NEC had a SrSO_2_ < 30% (p = 0.012).

**Table 2:** Cohort divided for exposure (SrSO2 above or below the 30% cut off)

	SrSO_2_ < 30% (n = 36)	SrSO_2_ ≥ 30% (n = 50)	P-value
Male, n(%)	25(69.4)	28(56.0)	0.26
Gestational age (weeks), median IQR	26.43(25.5-27-21)	25.71(24.71–27.14)	0.16
Section, n(%)	20(55.6)	25(50.0)	0.67
SGA, n(%)	8 (22.2)	8(16.0)	0.58
APGAR score at 5 min((median(IQR))	7(6–8)	7(6–8)	0.49
Birth weight (grams), SD	853(196)	817(280)	0.52
IVH Grade ≥ 2, n(%)	3(8.3)	8(16.0)	0.51
hsPDA, n(%)	21(58.3)	29(58.0)	1.00
Postnatal age of NIRS measurement, mean(SD)	3.84(0.88)	4.14(0.92)	0.14
Hb, median (IQR) at NIRS	144(140–157)	138(130–153)	0.07
> 50% breast milk, n (%)*	33(97.1)	48(96.0)	1.00
exclusive breast milk, n(%)	25(73.5)	37.(74.0)	1.00
Feeding amount > 30 ml/kg/d, n(%)	23(63.9)	35(70.0)	0.55
Mechanical ventilation, n(%)	14(38.9)	24(48.0)	0.51
SrSO2, median(IQR)	20.2(17.3-24.02)	48.6(38.11–61.59)	**0.00**
CrSO2,median (IQR)	75.7(68.6–79.6)	78.0(70.2–82.4)	0.40
SCOR, median(IQR)	0.27(0.23–0.33)	0.64(0.51–0.81)	**0.00**
Location : Swedish, n(%) Dutch, n(%)	16(44.4) 20(55.6)	28(56.0) 22(44.0)	0.38
NEC, n(%)	12 (33.3)	5(10.0)	**0.012**
surgical NEC, n(%)	10(27.8)	2(4.0)	**0.003**

Data are expressed as median (range), mean ± standard deviation for continuous variables or as number (percentage) for categorical variables. IVH: Intraventricular hemorrhage. hsPDA: hemodynamically significant patent ductus arteriosus. NEC: necrotizing enterocolitis. *: missing data for 2 patients

Sensitivity, specificity, negative and positive predictive values, and their 95% CIs were calculated (Table 3). The positive predictive value was 0.33 (95% CI 0.24–0.44) and the negative predictive value was 0.90 (95% CI 0.83–0.96).

**Table 3:** Sensitivity, specificity, positive and negative predictive value of SrSO2 < 30% in predicting NEC.

Cohort	Sensitivity (95%CI)	Specificity (95%CI)	PPV (95% CI)	NPV (95% CI)
**Combined cohort**	0.71 (0.47–0.88)	0.65 (0.54–0.77)	0.33 (0.24–0.44)	0.90 (0.83–0.96)
**Swedish cohort**	0.75 (0.38-1.00)	0.72 (0.58–0.86)	0.38 (0.23–0.56)	0.93 (0.84-1.00)
**Dutch cohort**	0.67 (0.33-1.00)	0.58 (0.39–0.76)	0.30 (0.17–0.44)	0.87 (0.75-1.00)

CI: confidence interval

**Fig. 2:**
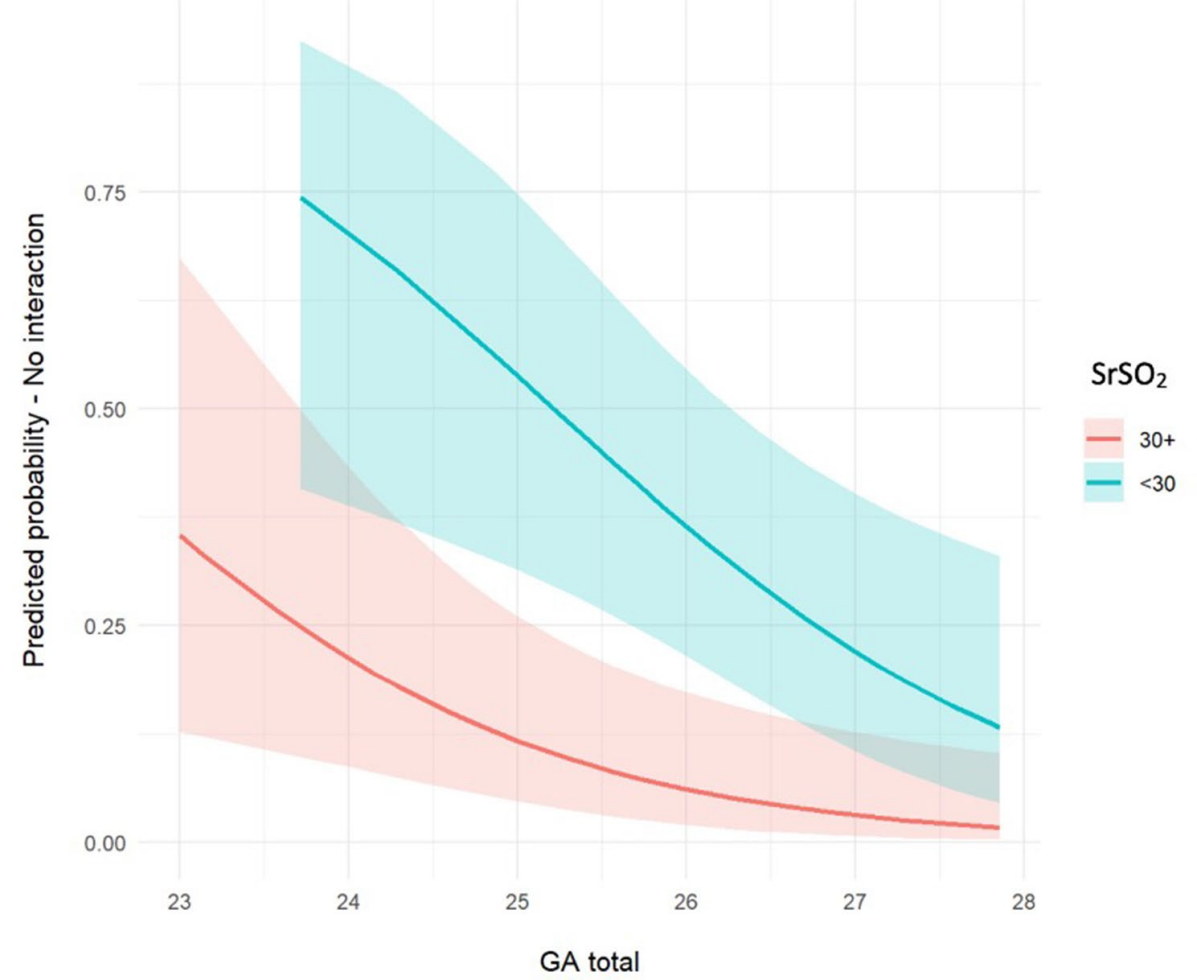
Graph showing the predicted probability of developing NEC in extremely preterm infants with SrSO2 < 30% (green) and in extremely preterm infants with SrSO2 ≥30% (in red)

The odds of developing NEC were 4.5 (95% CI 1.4–14.3) times higher in infants with SrSO2 < 30% compared to those with SrSO2 ≥ 30%. The odds ratio was still statistically significant after adjusting for gestational age and cohort: aOR 9.4 (95% CI 2.2–40.2) (Table 4). Because gestational age significantly changed the odds ratio, we plotted a graph to see how the predicted probability of developing NEC if SrSO2 < 30% changed with gestational age (Fig. 2). The association between SrSO2 < 30% and NEC seems to vary with changing gestational age. We therefore performed subgroup analyses and calculated the OR to develop NEC for infants born < 26 weeks and for those born ≥ 26 weeks of age (Table 4). In supplemental material 1, explanatory variables for infants who developed NEC and those who did not are shown. Univariate analyses are shown in supplemental material 2.

**Table 4:** Adjusted odds ratios with generalized linear model analysis. NEC was the dependent variable

	ODDS RATIO (95% confidence interval)	P-value
**SrSO**_**2**_ **< 30%, (adjusted for center)**	4.48 (1.40-14.31)	**0.011**
**SrSO**_**2**_ **< 30% (adjusted for center, gestational age)**	9.4 (2.20-40.23)	**0.003**
**SrSO**_**2**_ **< 30% (adjusted for center, in infants born < 26 weeks of age)**	13.53 (2.60-70.52)	**0.002**
**SrSO**_**2**_ **< 30% (adjusted for center, in infants born > = 26 weeks of age)**	2.15 (0.34–13.80)	0.418

## Discussion

We found in this multi-center study including extremely preterm infants that the cut off value of 30% for SrSO_2_, between day 2 and 6 after birth, has a high negative predictive value for NEC, but a rather poor positive predictive value. This indicates that this cut off for SrSO_2_ could help in identifying extremely preterm newborn infants with a low risk of NEC, but has limited value in positively predicting NEC development. The cut off value of 30% for SrSO_2_ is more discriminative for surgical NEC and in the smallest infants, born at a gestational age less than 26 weeks.

Despite an increasing amount of research, the role of NIRS as non-invasive monitoring of intestinal oxygenation in preterm infants is still uncertain and NIRS is generally not used as a clinical tool [12, 13]. Previous studies have also found lower SrSO_2_ in preterm infants before NEC onset [4, 14]. Patel et al. found a lower SrSO_2_ in the first week in preterm infants who later developed NEC compared to infants who did not develop NEC. Cortez et al. found a lower SrSO_2_ before NEC/SIP onset in a small study. However, they also found that in the first five days after birth, SrSO_2_ was similar between the infants who later developed NEC/SIP and the control infants. Moreover, Schat et al. found no lower SrSO_2_ in preterm infants between days 3 and 5 after birth, but only a trend of a lower SrSO_2_ in the 48 h before NEC development [5].

The predicted probability of developing NEC seems to vary with gestational age as the association between a SrSO_2_ < 30% and NEC was stronger in the more extreme preterm infants (< 26 weeks of gestation). It is known that SrSO_2_ varies with gestational and postnatal age [9], hence, gestational age is an important confounder in this study [15]. The reason for the fact that SrSO_2_ appears to be more predictive in the most extreme preterm infants remains unknown. Perhaps, in the complex multifactorial pathogenesis of preterm NEC, the contribution of immature splanchnic microcirculation may be larger for the smallest infants than for the older ones. An extremely immature splanchnic microcirculation has less capacity to maintain an adequate splanchnic oxygenation when needed, while slightly older infants may have a better capacity to maintain an adequate splanchnic oxygenation from becoming critically low [2, 16]. Another potential explanation would be that older infants have more stools which may affect the NIRS value increasing the absorption of NIRS photons [17] or due to the increased peristalsis that can affect the NIRS signal. It is also known that probiotics, which have been extensively studied as a prevention for NEC [18], do not work as well in infants with a birth weight of less than 1000 g [19, 20], meaning that preventing dysbiosis appears not to be enough in the smallest infants, where impaired microcirculation and ischemia might play a bigger role in NEC development.

A low splanchnic oxygenation is a proxy for impaired microcirculation or, less likely, increased intestinal oxygen consumption [21]. The use of splanchnic NIRS monitoring in the first week of life can lead to possible preventive measures, such as slower increasing of feeds, waiting for supplementations and a higher level of monitoring. Nevertheless, at the moment there is no evidence that all these strategies can actually decrease the risk of NEC or improve the outcome [22, 23]. Alternatively, being able to select a very high NEC risk population using NIRS would enable newly developed strategies to be tested in only this very high risk population. Not only that, it would also prevent possibly harmful strategies from being applied in this population.

The percentage of NEC-required surgery was very high in this population, probably due to the low gestational age of this combined cohort and the exclusion of uncertain NEC cases.

Although this study has some major strengths such as the combination of two cohorts from two different level three university hospitals, it also has some limitations such as the relatively small number of patients, differences between the two cohorts (i.e. different feeding strategies and umbilical catheters) and the use of only one single measurement. In the Dutch center, at the time of the study, we used to tape the umbilical catheter to the infraumbilical skin, which left only little space for the NIRS sensor, and unfortunately resulted in the exclusion of a number of small infants, potentially inducing selection bias. Another limitation of the study may be a substantial difference in the incidence of caesarean sections between both cohorts which may indicate a more active approach in the Swedish cohort, which was also reflected by the lower mean gestational age in this cohort. Finally, although the inclusion and exclusion criteria were similar between both cohorts, the Swedish cohort used an opt-in inclusion strategy while the Dutch cohort used an opt-out inclusion strategy for the study. Clearly all these differences could affect the outcome of the study. Even so, we did not detect a difference in SrSO_2_ between the two centers. Moreover, we corrected for the potential bias of center in the regression analysis.

The cut off was previously calculated from the Swedish cohort, but being aware that the results from small cohort carry uncertainty and less external validity, we needed to test it again in a combined cohort to increase the validity of the results and to study the association between SrSO_2_ and surgical NEC with increased numbers.

There are also limitations related to NIRS measurements and technology. Short measurements that are not repeated as well as artefacts (such as movements, air, and stools) are the two most important ones. We opted for short measurement durations because it was easier to ensure the quality of the SrSO_2_ in the smallest infants and to better estimate when artefacts were caused by movements.

In conclusion, using a cut off of 30% for splanchnic oxygenation measured in the first week in extremely preterm infants appeared to be clinically useful only in identifying infants who will not develop NEC. Finally extremely preterm infants with splanchnic oxygenation below 30% in the first week of life carry higher odds of developing NEC, especially if born before 26 weeks of gestation, supporting the contribution of gut hypoxia to NEC development in extremely preterm born infants.

## Electronic supplementary material

Below is the link to the electronic supplementary material.

Supplementary Material 1

Supplementary Material 2

## Data Availability

The datasets generated during and analysed during the current study are available from the corresponding author on reasonable request.
