# Supplementary material for: Clinical usefulness of splanchnic oxygenation in predicting necrotizing enterocolitis in extremely preterm infants: a cohort study
Source: BMC Pediatr. 2023 Jul 1;23:336. doi: 10.1186/s12887-023-04145-4 (PMC10314466; doi:10.1186/s12887-023-04145-4)
Supplement: Supplementary file 1 — Supplementary Material 1 [file 12887_2023_4145_MOESM1_ESM.docx]

***Supplemental material 1:*** *Explanatory variables divided by outcomes*

|  | No NEC (69) | NEC(17) | p-value |
| --- | --- | --- | --- |
| Male,n(%) | 41 ( 59.42) | 12 ( 70.6) | 0.40 |
| Gestational age (median [IQR]) | 26.43 [25.28-27.29] | 25.43 [25.00, 26.14] | 0.023 |
| Section n,(%) | 39 ( 56.52) | 6 ( 35.3) | 0.18 |
| APGAR 5min (median ((IQR)) | 6(5-8) | 7(6-8) | 0.25 |
| SGA(%) | 14 ( 20.29) | 2 ( 11.76) | 0.73 |
| Birthweight_g (mean (SD)) | 846(262) | 777(167) | 0.31 |
| IVH Grade >2, n(%) | 8(11.76) | 2(11.76) | 1.00 |
| hsPDA, n(%) | 39(56.52) | 11(64.7) | 0.59 |
| Postnatal age of NIRS measurement (mean (SD)) | 3.99 (0.91) | 4.11 (0.91) | 0.61 |
| Hb,median( IQR) at NIRS | 142(131-153) | 145(136-159) | 0.189 |
| Mechanical ventilation, n(%) | 29(42.03) | 9(52.94) | 0.53 |
| feeding >30 ml/kg/day) | 45(65.22) | 13(76.47) | 0.38 |
| exclusive breast milk, n(%) | 52(76.47)) | 10(62.5) | 0.34 |
| >50% breast milk,n (%) | 15(93.75) | 66(97.06) | 0.35 |
| SrSO_2_<30% | 24 ( 34.8) | 12 ( 70.6) | 0.012 |
| SrSO_2_ (median [IQR]) | 37.1(23.3- 52.4) | 23.02 (20.21, 39.51) | 0.12 |
| CrSO_2_ (median [IQR]) | 77.3(72.1-85.8) | 75.5(70.5-81-2) | 0.51 |
| SCOR(median [IQR]) | 0.49(0.32-0.74) | 0.31(0.26-0.52) | 0.06 |
| Cohort (%) Dutch | 36 ( 52.17) | 9 ( 52.9) | 0.71 |
| Swedish | 33 ( 47.83) | 8 ( 47.1) |  |

# 
