# Supplementary material for: Clinical usefulness of splanchnic oxygenation in predicting necrotizing enterocolitis in extremely preterm infants: a cohort study
Source: BMC Pediatr. 2023 Jul 1;23:336. doi: 10.1186/s12887-023-04145-4 (PMC10314466; doi:10.1186/s12887-023-04145-4)
Supplement: Supplementary file 2 — Supplementary Material 2 [file 12887_2023_4145_MOESM2_ESM.docx]

***Supplemental material 2:*** *Univariate analysis, outcome NEC*

|  | **OR ( 95% CI)** | **p value** |
| --- | --- | --- |
| Gestational age | 0.66(0.44-0.99) | **0.046** |
| Location | 1.23(0.42-3.55) | 0.71 |
| Section | 0.42(0.139-1.26) | 0.12 |
| Birth weight | 0.16(0.011-2.40) | 0.19 |
| Breast milk exclusively | 0.51(0.16-1.63) | 0.258 |
| 50% breast milk | 0.45(0.38-5.34) | 0.531 |
| Hemoglobin | 1.04 (1.00-1.08) | 0.074 |
| Patent Ductus Arteriosus | 1.41(0.47-4.24) | 0.541 |
| Feed >30 ml/kg/d | 1.73(0.51-5.90) | 0.379 |
| SGA | 0.52(0.11-2.56) | 0.425 |
| SrSO2<30% | 4.50(1.42-14.28) | **0.011** |
| SrSO2 continuous | 0.98(0.94-1.01) | 0.164 |
